# Abnormal expression of miR-3653-3p, caspase 1, IL-1β in peripheral blood of schizophrenia

**DOI:** 10.1186/s12888-023-05182-0

**Published:** 2023-11-09

**Authors:** Xin-ling Zhao, Yi-lin Liu, Qing Long, Yun-qiao Zhang, Xu You, Ze-yi Guo, Xiang Cao, Lei Yu, Fu-yi Qin, Zhao-wei Teng, Yong Zeng

**Affiliations:** 1 Department of Psychiatry, The Second Affiliated Hospital of Kunming Medical University, Kunming, Yunnan Province China; 2 Psychiatric Ward, Honghe Second People’s Hospital, Honghe, Yunnan Province China; 3 Central Laboratory of the Second Affiliated Hospital of Kunming Medical University, Kunming, Yunnan Province China

**Keywords:** Schizophrenia, IL-1β, NLRP3, miR-3653-3p, Caspase 1, qPCR

## Abstract

Schizophrenia (SCZ) is a chronic, highly relapsing, severe mental disorder with an unclear etiology. Cytokine-mediated neuroimmune abnormalities have been repeatedly revealed. IL-1β was reported to play a vital role in expanding the inflammatory response. However, the underlying molecular mechanism is poorly understood. In this study, we found that miR-3653-3p with the NLRP3 binding site in Targetscan was differentially expressed in miRNA high-throughput sequencing in schizophrenia (SCZ), and indeed, its downregulation in SCZ peripheral blood was also verified by RT-qPCR (*P*-value = 0.015). Furthermore, we found that the mRNAs of caspase 1 and IL-1β are elevated in people who suffer from SCZ (*P* = 0.044 and *P* = 0.001, respectively). Moreover, the interaction of NLRP3, Caspase1, and IL-1β was found in the peripheral blood of patients with SCZ. The expression level of miR-3653-3p was negatively correlated with NLRP3 and IL-1β mRNA contents (*r* = 0.487, *P* = 0.04 and *r* = 0.508, *P* = 0.037, respectively). NLRP3 mRNA was positively correlated with caspase1 mRNA. Meanwhile, the expression of miR-3653-3p was also negatively correlated with negative symptom subscores of PANSS (*r* = 0.450, *P* = 0.046). IL-1β mRNA is positively correlated with the total scores of PANSS (*r* = 0.690, *P* = 0.002) and the sub-scores of general psychopathology of PANSS (*r* = 0.583, *P* = 0.014). Additionally, a significant positive relationship exists between IL-1β and the total duration (*r* = 0.638, *P* = 0.006). We found that the combination of miR-3653-3p, caspase 1, and IL-1β have better diagnostic values. The results indicate that miR-3653-3p, caspase 1, and IL-1β can potentially be biomarkers of SCZ, identifying negative symptoms or a chronic course. A further understanding of the involvement of IL-1β in SCZ may be a crucial molecular effector for the chronic course to intervene.

**Supplementary Information:**

The online version contains supplementary material available at 10.1186/s12888-023-05182-0.

## Introduction

Schizophrenia is a heterogeneous syndrome involving emotion, thinking, cognition, and behavior. It is characterized by severe symptoms, a chronic course, and high recurrence rates, making it among the top ten disabling diseases in the world [1]. Cytokine-mediated activation of neuroimmune cells in schizophrenia has been continuously revealed [2–6]. Among them, IL-1β has repeatedly been shown to be elevated in the peripheral blood and cerebrospinal fluid of patients with schizophrenia [7–10]. IL-1β and its single nucleotide polymorphisms are implicated in white matter and gray matter volume abnormalities in schizophrenia [11, 12]. In animal experiments, injections of IL-1β induce psychotic-like symptoms [13]. It is considered an attractive candidate for studying brain function in healthy individuals and various mental disorders [14]. However, there are few reports on the small molecule regulation mechanism of IL-1β. Previously, differentially expressed prefrontal cortex NLRP3, Caspase 1, and IL-1β have been reported in post-mortem brains of schizophrenia patients [15]. In cellular experiments, NLRP3 was inhibited, and downstream caspase 1 and IL-1β expression were down-regulated; microglia activation decreased [16]. Intriguingly, NLRP3/Caspase 1/IL-1β is also involved in the classical pyroptosis pathway, in which IL-1β and IL-18 are activated, and the inflammation is amplified, which may contribute to the pathogenesis of schizophrenia [17]. The role of NLRP3, Caspase 1, and IL-1β in the pathological process of schizophrenia deserves further investigation.

miRNAs are classical post-transcriptional modifications involved in cell proliferation, immune regulation, and cell death by negatively regulating target genes [18, 19]. As stable and quantifiable star vectors, they have been identified as having great potential as biomarkers for complex central disorders [19, 20]. Schizophrenia has been revealed to have inflammatory-related miRNA and its target gene expression abnormalities [21]. miRNAs may regulate NLRP3-related inflammatory pathways in mental disorders [22]. We screened miRNAs with NLRP3 binding sites by miRNA high-throughput sequencing and Targetscan. Finally, we detected miR-3653-3p and NLRP3, Caspase 1 and IL-1β mRNA and explored the relationship between miR-3653-3p and NLRP3, Caspase 1, IL-1β in peripheral blood of schizophrenia; we look forward to searching for stable biomarkers of schizophrenia in peripheral blood.

## Materials and methods

### Participants and samples

All Participants were recruited from the Second People’s Hospital of Honghe Prefecture, Yunnan Province. Schizophrenia diagnosis was based on the Diagnostic and Statistical Manual of Mental Disorders (Fifth Edition) (DSM-V). The patients were recruited from those diagnosed with schizophrenia and admitted to the emergency department from June to December 2022. HCs were enrolled in the physical examination center during the same period in healthy adults. Subjects were excluded for the following: (1) patients with a history of mental retardation, epilepsy, encephalitis, and other organic brain diseases or other neurological diseases; (2) patients who have been diagnosed with other mental disorders or have taken antipsychotic drugs in the past month; (3) patients with systemic diseases such as immune, endocrine, or metabolic disorders; (4) alcohol or other substance abuse or dependence; (5) those at risk of killing themselves or harming others. A total of 20 people were recruited into the study group, 8 of whom completed 12 weeks of follow-up.

The control group was enrolled according to the following criteria: No present mental illness, no history of mental illness, and no family history of mental illness. The exclusion criteria were the same as in the patient group. A total of 15 healthy controls were recruited.

### miRNA high-throughput sequencing

RNA was extracted by Trizol method, and then RNA purity was detected by NanoDrop 2000 (Thermo Fisher Scientific, Inc.). RNA integrity was detected with Agilent 2100 Bioanalyzer (Agilent Technologies GmbH). The total RNA was then subjected to gel electrophoresis to produce small RNAs, with the 5' end and 3' end joined. Small RNA libraries were constructed by RT-qPCR. And then, the constructed libraries were tested for quality and yield using Agilent 2100 and ABI StepOnePlus Real-Time PCR System (Thermo Fisher Scientific). Sequencing was performed using illumina HiSeq2000 (Illumina, inc.). All procedures are strictly in accordance with the manufacturer’s protocol (Gidio Biotechnology co., ltd.).

### miRNAs screening for NLRP3 binding sites

In the miRNA expression matrix, the *P* value and |LogFC| value combination of screening differentially expressed miRNAs is a widely used method. In this study, the differential miRNA was set at *P* < 0.05 and |LogFC|> 1 or |LogFC|< -1; Then, we screened miRNAs with NLRP3 binding sites in TargetScan 8.0 (https://www.targetscan.org/vert_80/), an open database that is widely used. We performed Venn diagrams for differential miRNAs from high-throughput sequencing and miRNAs from TargetScan. We screened out miR-3653-3p and then validated the expression of miR-3653-3p in blood samples.

### Blood samples processing and total RNA extraction

2 ml of whole blood was collected from 20 patients and 15 healthy subjects. The whole blood and red cell lysate were mixed at a ratio of 1:3. After standing (5 min) and centrifugation (4000 r/min, 5 min), the supernatant was removed. After cell precipitation remained, 1 ml of Trizol was added, and the mixture was frozen in the refrigerator at -80 °C. When extracting total RNA, 200ul of chloroform was added after the frozen sample was melted at room temperature, thoroughly mixed, and allowed to stand for 10 min. After centrifugation (4 °C, 12000 g, 15 min), the supernatant was collected and put into another tube (pay attention not to suck into the second layer), and isopropyl alcohol was added into it at a ratio of 1:1. After standing in the -20 °C refrigerator for 20 min, centrifuge again (4 °C, 12000 g, 10 min). Total RNA pellets were cleaned with 75% ethanol, centrifuged at 7500 g at 4℃ for 10 min, removed supernatants, and dried at room temperature. Finally, total RNA was dissolved in 20µL of enzyme-free water and bathed in a 55° water bath for 10 min. ND2000 nucleic acid quantifier was used for preliminary quantification.

### cDNA synthesis

After the total RNA extraction, according to the manufacturer’s solution, we take the PrimeScript ™ RT reagent Kit with gDNA Eraser (Perfect Real Time) (Takara, Biotech) to synthesize cDNA of miRNA. Use TransScript All-in-One First-Strand cDNA Synthesis SuperMix for qPCR (One-Step gDNA Removal) (TransGen, Biotech) to synthesize cDNA of the target gene. The primer sequence is shown in the supplementary information.

### RT-qPCR

TransScript II Green One-Step qRT-qPCR SuperMix was used to detect the expression levels of miR-3653-3p, NLRP3, Caspase 1, and IL-1β in schizophrenia and healthy controls. RT-qPCR in quantitative PCR response amplifier (Roche LightCycler480 real-time fluorescence quantitative PCR), The reaction system includes 2xPerfectStart Green One-Step qPCR SuperMix, TranScript II Green One-Step RT/RI Enzyme Mix, Forward Primer, and Reverse Primer. The results were normalized using U6 for miRNA, and the expression level of miRNA was calculated using 2^−ΔΔct^ method. After treatment with antipsychotic drugs, the expression levels of miR-3653-3p, NLRP3, Caspase 1, and IL-1β were remeasured as described above. 

### PANSS scale assessment

Two attending psychiatrists who had been trained and met the requirements of the scale jointly conducted psychiatric examinations for the patients, and evaluated and scored the patients based on the relevant information provided by their families (the Kappa coefficient is 0.84).

### Statistical analysis

SPSS 21.0 was used for statistical analysis. GraphPad Prism 9.4.1 was used for making charts. miR-3653-3p, NLRP3 mRNA, caspase 1 mRNA, and IL-1β mRNA in the patient and healthy control groups were all converted by square root. The comparison of miR-3653-3p, NLRP3 mRNA between the two groups was performed by Mann–whitney tests. The comparison of caspase 1 mRNA, IL-1βmRNA was performed by independent sample *t*-test. *χ*^*2*^ test was used for sex and t test was used for age. The expression levels of miR-3653-3p, NLRP3, and Caspase 1 before and after treatment were compared by paired rank sum test. The expression levels of IL-1β before and after treatment were tested by paired t-test. Spearman correlation analysis was used to determine the relationships between miR-3653-3p, NLRP3, and PANSS scores in the patient group. The relationships between caspase 1, IL-1β, and PANSS scores were analyzed by Pearson Correlation Analysis. All correlation analyses were performed with Bonferroni correction. A receiver operating characteristic curve was completed to confirm the diagnostic value of miR-3653-3p、caspase 1、IL-1β. The test level α = 0.05 was used for bilateral test.

## Results

### Basic information

Both the cases and the controls were Han, and the mean ages of the two groups were 36.5 ± 11.993 and 33.692 ± 11.506. The dates showed no statistical differences in age and gender between the two groups. See Table 1.

**Table 1:** Demographic data for the cases and healthy controls

	Cases (*n* = 25)	Controls (*n* = 14)	*P*-value ^a^
Sex			0.065
Male	16(80%)	6(46.2%)	
Female	4(20%)	7(53.8%)	
Age (years)	36.5 ± 11.993	33.692 ± 11.506	0.509
PANSS			
Total	85.85 ± 7.731	–	–
Positive Symptom	21.35 ± 2.109	–	–
Negative Symptom	22.4 ± 3.633	–	–
Duration of illness	7(years)	–	–

Values are expressed as n (%) or the mean ± standard deviation

^a^ A *t* -test was performed for quantitative variables and a χ2 test was performed for categorical variables

### miRNA selection

**Fig. 1:**
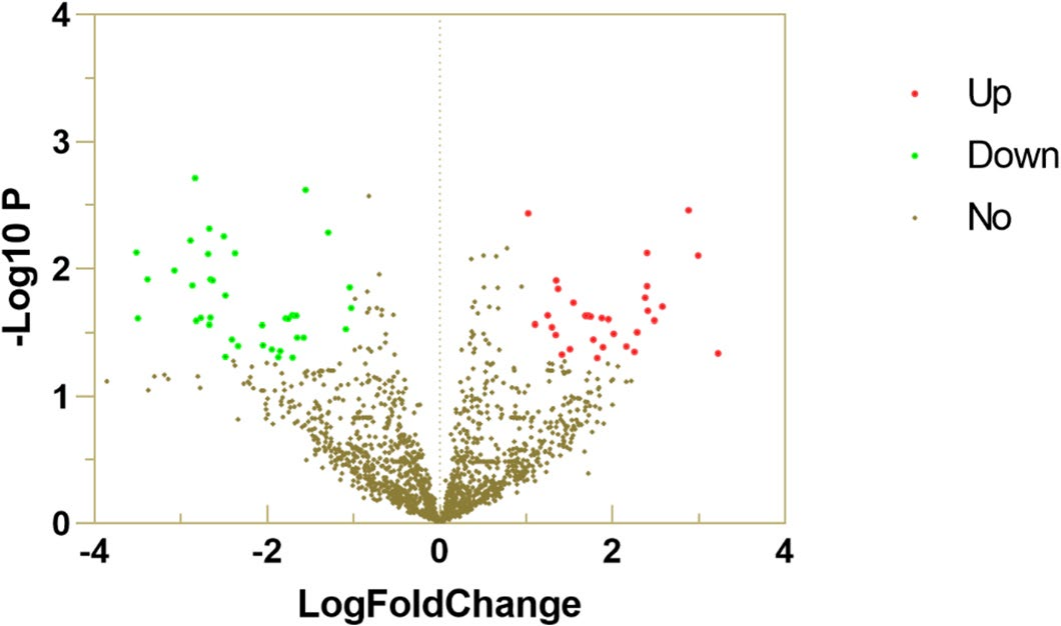
Volcano plot of differentially expressed miRNA

**Fig. 2:**
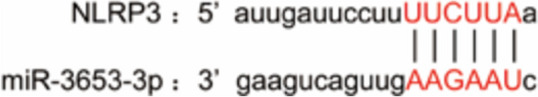
miR-3653-3p interaction site with NLRP3

We found 41 miRNAs with lower expression and 35 miRNAs with higher expression. See Table 2. | LogFC | was used to make a volcano map. See Fig. 1. The interaction sites between miR-3653-3p and NLRP3 are shown in Fig. 2. The details of NLRP3’s interaction with miR-3653-3p are shown in Table 3.

**Table 2:** Differentially expressed miRNAs in high throughput sequencing of peripheral blood

miRNA	logFC	*P*.Value	q-value	direction of change
hsa-miR-122-5p	4.538	0.000	0.887	up
hsa-miR-4762-5p	2.883	0.003	0.887	up
miR-12215	1.023	0.004	0.887	up
hsa-miR-580-5p	2.403	0.007	0.887	up
hsa-miR-490-3p	2.994	0.008	0.887	up
hsa-miR-5683	1.350	0.012	0.887	up
hsa-miR-6733-5p	2.402	0.014	0.887	up
hsa-miR-486-5p	1.373	0.014	0.887	up
hsa-miR-92b-5p	2.380	0.017	0.887	up
hsa-miR-4732-5p	1.551	0.019	0.887	up
hsa-miR-211-5p	2.581	0.020	0.887	up
miR-6861	2.412	0.021	0.887	up
hsa-miR-96-5p	1.250	0.023	0.887	up
hsa-miR-4777-3p	1.689	0.023	0.887	up
miR-23	1.721	0.023	0.887	up
miR-10174	1.721	0.023	0.887	up
miR-939	1.697	0.023	0.887	up
hsa-miR-6812-3p	1.749	0.024	0.887	up
miR-1587	1.880	0.024	0.887	up
hsa-miR-135b-5p	1.953	0.025	0.887	up
hsa-miR-3127-5p	2.488	0.025	0.887	up
hsa-miR-3688-3p	1.107	0.027	0.887	up
hsa-miR-4685-3p	1.302	0.029	0.887	up
hsa-miR-376b-5p	2.288	0.031	0.887	up
hsa-miR-376c-5p	2.288	0.031	0.887	up
miR-2779	2.016	0.032	0.887	up
hsa-miR-4732-3p	1.343	0.033	0.887	up
novel-m0133-3p	1.782	0.036	0.887	up
novel-m0119-3p	2.164	0.041	0.887	up
hsa-miR-610	1.890	0.041	0.887	up
hsa-miR-548p	1.508	0.043	0.887	up
novel-m0142-5p	2.256	0.045	0.887	up
hsa-miR-124-3p	3.222	0.046	0.887	up
hsa-miR-451a	1.416	0.047	0.887	up
hsa-miR-6743-3p	1.825	0.050	0.887	up
hsa-miR-3653-3p	-4.888	0.000	0.000	down
miR-1278	-2.835	0.002	0.887	down
miR-3971	-1.555	0.002	0.887	down
novel-m0108-3p	-2.669	0.005	0.887	down
miR-12135	-1.293	0.005	0.887	down
miR-150	-2.500	0.006	0.887	down
hsa-miR-34c-3p	-2.889	0.006	0.887	down
miR-618	-3.512	0.007	0.887	down
hsa-miR-155-3p	-2.373	0.008	0.887	down
miR-940	-2.680	0.008	0.887	down
hsa-miR-642b-5p	-3.073	0.010	0.887	down
miR-4772	-3.386	0.012	0.887	down
miR-322	-2.655	0.012	0.887	down
miR-1287	-2.629	0.012	0.887	down
miR-4772	-2.865	0.013	0.887	down
miR-141	-1.043	0.014	0.887	down
miR-5127	-2.481	0.016	0.887	down
let-7	-1.027	0.020	0.887	down
miR-83	-1.711	0.023	0.887	down
miR-3194	-1.680	0.023	0.887	down
miR-6724	-1.659	0.023	0.887	down
miR-95	-2.657	0.024	0.887	down
miR-7	-2.765	0.024	0.887	down
hsa-miR-99a-3p	-1.784	0.025	0.887	down
miR-465	-3.494	0.025	0.887	down
miR-3064	-1.753	0.025	0.887	down
miR-881	-2.818	0.026	0.887	down
hsa-miR-5701	-2.667	0.028	0.887	down
miR-3605	-2.057	0.028	0.887	down
miR-145	-1.085	0.030	0.887	down
miR-324	-1.652	0.034	0.887	down
hsa-miR-3607-3p	-1.577	0.035	0.887	down
miR-3967	-2.409	0.036	0.887	down
hsa-miR-4799-5p	-2.045	0.040	0.887	down
novel-m0018-5p	-2.335	0.040	0.887	down
novel-m0019-5p	-2.335	0.040	0.887	down
miR-140-x	-1.945	0.043	0.887	down
hsa-miR-6804-3p	-1.847	0.044	0.887	down
hsa-miR-4433a-3p	-2.480	0.049	0.887	down
hsa-miR-449a	-1.871	0.049	0.887	down
miR-32	-1.706	0.050	0.887	down

**Table 3:** Details of the interaction between NLRP3 and miR-3653-3p

Position	Site type	Context + + score	Context + + score percentile
588–594 of NLRP3 3' UTR	7mer-A1	-0.15	95

### RT-qPCR data analysis

**Fig. 3:**
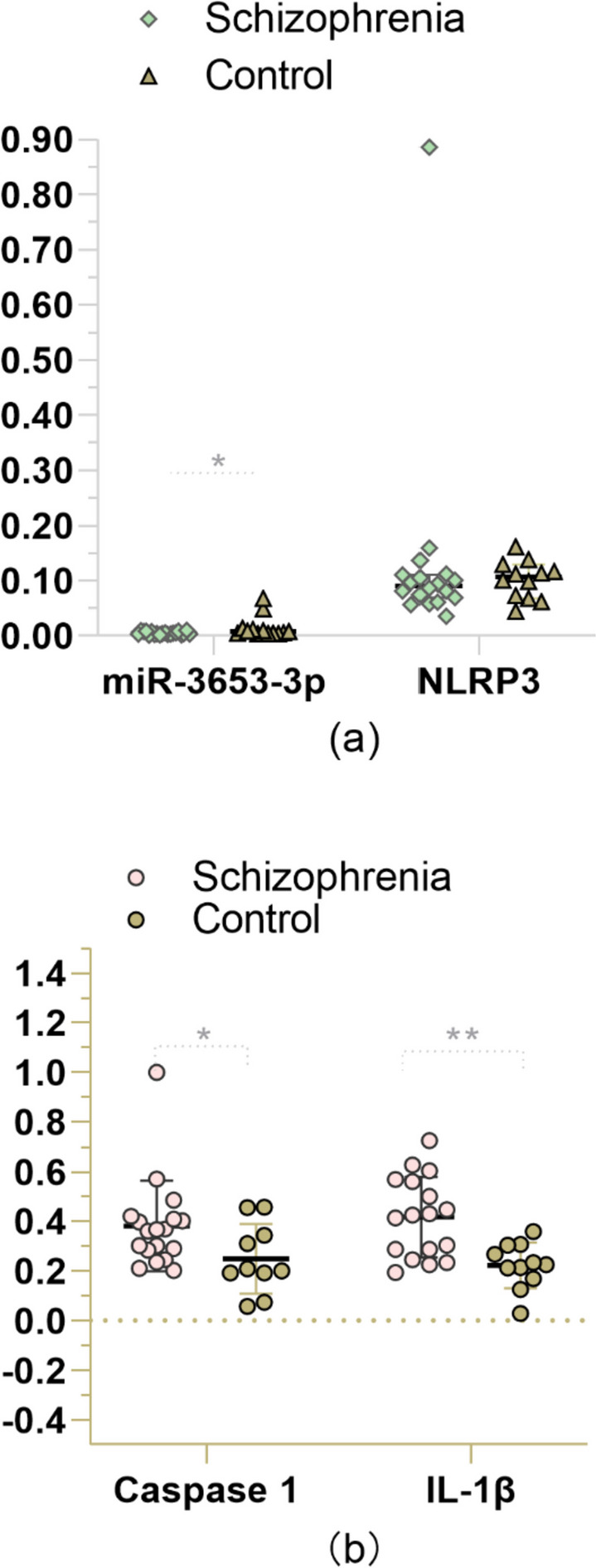
Comparisons of miR-3653-3p、NLRP3、Caspase 1 and IL-1β relative expression between the cases and the controls. **a** Scatter plot with median and 95% confidence interval between the case and control; **b** Scatter plot with mean and standard deviation

The results show that miR-653-3p in the study group is lower than that in the control group (Z = -2.433, *P* = 0.015); the expression levels of Caspase-1 in the study group were higher than those in the healthy group (*F* = 0.000, *P* = 0.044), and the expression levels of IL-1β in the study group were significantly higher than those in the control group (*F* = 5.325, *P* = 0.001). See Table 4 and Fig. 3. After treatment with antipsychotic drugs, the expressions of miR-3653-3p, NLRP3, Caspase 1, and IL-1β were re-detected. We found no statistically significant changes in all four. See Table 5.

**Table 4:** Comparison of miR-3653-3p、NLRP3、Caspase 1 and IL-1β expression in peripheral blood between the cases and healthy controls

	Cases	Controls	F/Z	*P*-value ^a,b^
miR-3653-3p	0.004	0.008	-2.433	0.015*
NLRP3	0.090	0.101 ± 0.034	-0.889	0.374
Caspase 1	0.381 ± 0.182	0.249 ± 0.140	0.000	0.044*
IL-1β	0.416 ± 0.162	0.222 ± 0.091	5.325	0.001**

^a^ miR-3653-3p and NLRP3 were converted and tested by Mann–Whitney U; Caspase 1 mRNA and IL-1β mRNA were transformed and* t* -test were performed

^b^* *P* < 0.05; ** *P* < 0.01

**Table 5:** Comparisons of miR-3653-3p、NLRP3、Caspase 1 and IL-1β expression in peripheral blood of the cases before and after drug treatment

	Before	After	Chi-square	*P*-value
miR-3653-3p	0.006	0.005	0.500	0.480
NLRP3	0.059	0.059	1.800	0.180
Caspase 1	0.241	0.206	0.000	1.000
IL-1β	0.304	0.209	1.000	0.317

The expression levels of the four before and after drug treatment were all transformed and performed by non-parametric tests

### Correlation analysis

**Fig. 4:**
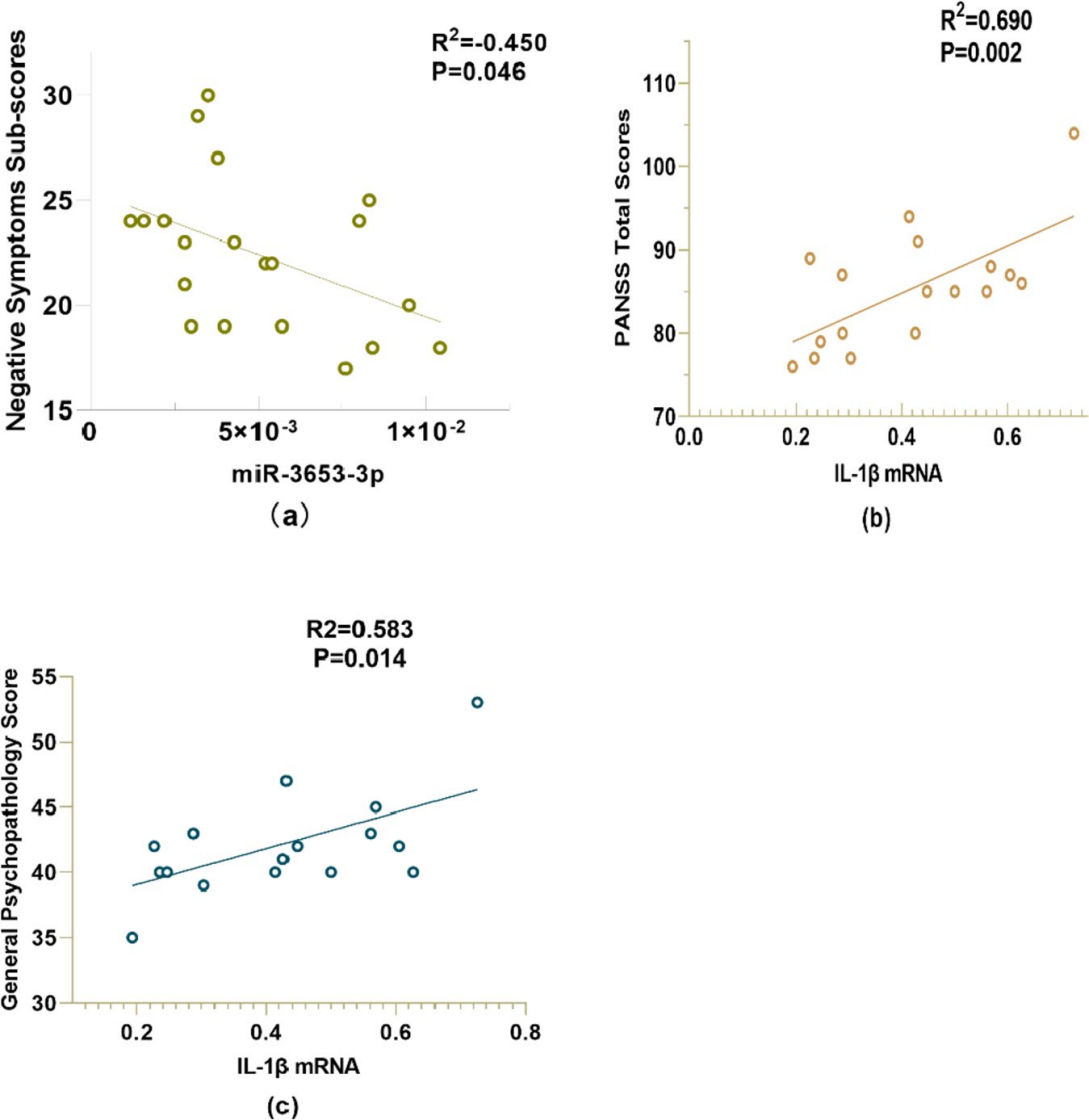
Correlation of miR-3653-3p, IL-1β with total score and subscore of PANSS. **a** Correlation between miR-3653-3p and negative symptom score; **b** Correlation between IL-1β and PANSS total score; **c** Correlation between IL-1β and general psychopathology score

**Fig. 5:**
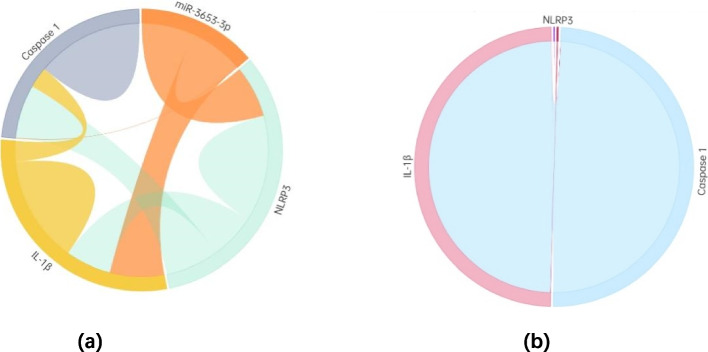
Comparisons of correlations between miR-3653-3p, NLRP3, Caspase1 and IL-1β between the cases and the controls

We analyzed the correlations between the transformed miR-3653-3p, NLRP3 mRNA, Caspase 1 mRNA, IL-1β mRNA and the total scores of PANSS, positive symptoms, negative symptoms, and general psychopathological sub-scores. We also analyzed the interaction of the four. We found that miR-3653-3p was negatively correlated with PANSS negative symptoms by Spearman correlation analysis (*r* = -0.450, *P* = 0.046). See Fig. 4a. IL-1β mRNA was positively correlated with the total scores of PANSS (*r* = 0.690, *P* = 0.002) and the general psychopathological sub-scores of PANSS (*r* = 0.583, *P* = 0.014) by Pearson Correlation Analysis. See Fig. 4b, c. Spearman correlation analysis showed that miR-3653-3p was negatively correlated with NLRP3 (*r* = -0.487, *P* = 0.04), and miR-3653-3p was negatively correlated with IL-1β (*r* = -0.508, *P* = 0.037); NLRP3 was positively correlated with caspase 1 (*r* = 0.501, *P* = 0.034) in the patients. See Fig. 5a. In the control group, we only found that caspase 1 was positively correlated with IL-1β (*r* = 0.725, *P* = 0.027). See Fig. 5b. After the Bonferroni correction, we concluded that only the correlation between IL-1β and PANSS total score was statistically significant (a = 0.05/22 = 0.0023).

We also analyzed the relationships between the converted miR-3653-3p, NLRP3, Caspase 1, IL-1β and the total course of the disease and found that IL-1β was positively correlated with the duration of illness (*r* = 0.638, *P* = 0.006). See Table 6.

**Table 6:** The relationships between the duration of schizophrenia and miR-3653-3p, NLRP3, Caspase 1, IL-1β

	miR-3653-3p	NLRP3	Caspase 1	IL-1β
Duration of illness	-0.098	-0.006	-0.370	0.638
P	0.681	0.981	0.130	0.006**

miR-3653-3p、NLRP3、Caspase 1、IL-1β were converted; Spearman correlation analysis were performed. According to the Bonferroni correction, a = 0.05/4 = 0.0125; **P<0.01

### ROC curve analysis

**Fig. 6:**
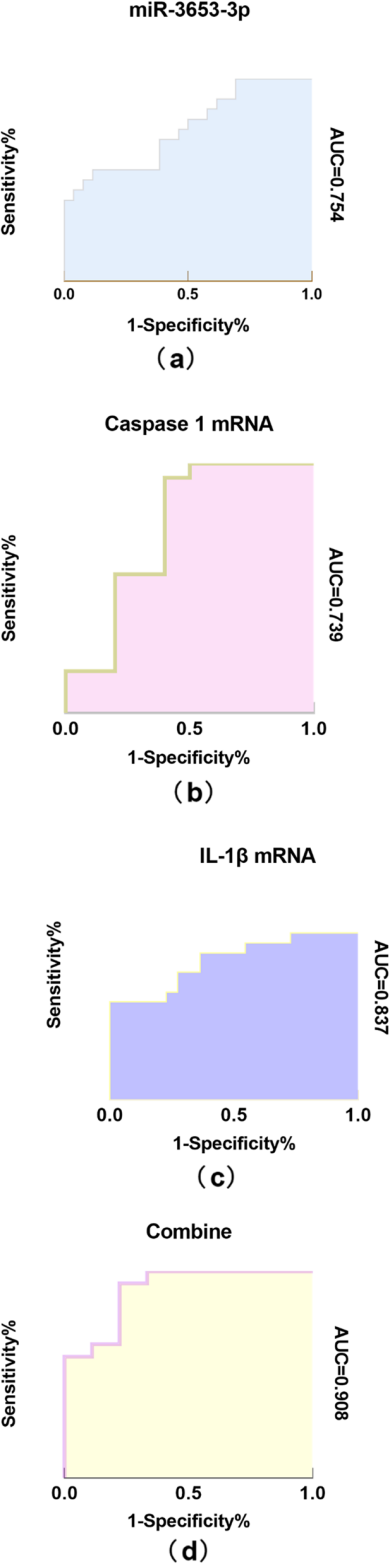
Diagnostic evaluation of miR-3653-3p、Caspase1、IL-1β and combine of them. ROC curves of **A** miR-3653-3p and **B** Caspase1, and **C** IL-1β, and **D** their combined roc curve. ROC, receiver operating characteristic; AUC, area under the curve; miR, microRNA

ROC curve analysis was completed to assess the potential of miR-3653-3p, caspase1, and IL1β expression levels confirmed by RT-qPCR as biomarkers for distinguishing patients with schizophrenia from healthy controls. As shown in Fig. 6, the AUC for miR-3653-3p was 0.754 (95% CI, 0.5892–0.9185; *P* = 0.015; Fig. 6A), and the best cutoff value was 0.423 (specificity, 0.923; sensitivity, 0.50). The AUC for caspase 1 was 0.739 (95% CI, 0.5181–0.9596; *P* = 0.039; Fig. 6B), and the best cutoff value was 0.544 (specificity, 0.60; sensitivity, 0.944). The AUC for IL-1β was 0.837 (95% CI, 0.6906–0.9823; *P* = 0.003; Fig. 6C), the best cutoff value was 0.518 (specificity, 0.636; sensitivity, 0.882). The AUC for the combination of the three was able to clearly distinguish patients from healthy controls with an AUC of 0.908 (95% CI, 0.787–1.0; *P* = 0.001; Fig. 6D), the best cutoff value was 0.51 (specificity, 0.778; sensitivity, 0.941).

## Discussion

Previously NLRP3/Caspase1/IL-1β has been implicated in central immune and neurodegenerative diseases [15, 23]. Our results show that the expressions of miR-3653-3p in the peripheral blood of patients with acute schizophrenia were lower, which was first reported in schizophrenia, and the relative expressions of caspase 1 mRNA and IL-1β mRNA were all elevated. And the lower expression of miR-3653-3p, the higher the IL-1β mRNA. The lower expression of miR-3653-3p, the higher expression of NLRP3 mRNA. The NLRP3 showed an increasing trend, and caspase 1 also increased correspondingly. The simultaneous detection of all three has not previously been reported in the peripheral blood of schizophrenia patients. Thus, our study results also indicated that miR-3653-3p could be involved in the expression of NLRP3, caspase 1, and IL-1β, though NLRP3 was not statistically different between patients with schizophrenia and the controls. It echoes previous postmortem reports of schizophrenia [15]. Besides, we found that miR-3653-3p may be associated with negative symptoms of schizophrenia, and IL-1β levels may be positively associated with schizophrenia symptoms, consistent with previous reports [7, 8]. To some extent, this could not directly reflect the negative regulatory function of miR-3653-3p and ensure whether NLRP3/Caspase1/IL-1β is a regulatory axis in schizophrenia, but this indicates miR-3653-3p, NLRP3, caspase 1 and IL-1β are closely related in schizophrenia.

In previous studies, as a crucial proinflammatory cytokine, IL-1β was recognized as a signature molecule and differentially expressed in peripheral blood and cerebrospinal fluid of schizophrenia [8, 10]; whether IL-1β is a cause or an effect of schizophrenia is unknown. Nevertheless, previously IL-1β in peripheral blood has been revealed to likely exert their effects on the brain via primary afferent neurons or from the blood through periventricular organs and the choroid plexus [24]. Intriguingly, IL-1β is thought to be the result of enlarged inflammation in the mother, but as a cause of schizophrenia in the offspring, it has been conjectured that blood–brain barrier damage may occur in the early years or even in the fetal periods, then peripheral inflammation interacts with central immune activation in schizophrenia [4, 25, 26]. Furthermore, not only IL-1β was involved in abnormalities in the volume of white matter and gray matter in the brain of schizophrenia [11, 27], but also associated with speech fluency and decreased Broca region volume in schizophrenia [28]. Most importantly, IL-1β was also revealed to be essential for hippocampal-dependent learning and memory via microglia [29–31]. Moreover, IL-1β has been concluded to be the final effector, contributing to inflammation-related cognitive dysfunction [5, 32, 33], while cognitive impairment is considered along with schizophrenia in its early stages [34]. At last, we found an association between IL-1β and the chronic course of schizophrenia; this indicates that IL-1β is involved in the chronic course of schizophrenia. Significant increases in IL-1β during acute exacerbation of chronic schizophrenia have been reported before [12, 35]. Therefore, the relationship between IL-1β and the pathological mechanism of schizophrenia deserves further exploration. IL- 1β has previously been speculated to damage neurons directly or indirectly, leading to cell death [36]. It is worth mentioning that IL-1β release is once considered a marker of cell death [37]. Caspase 1/IL-1β is also an important pathway of pyroptosis [16, 38], which is a programmed cell death associated with inflammatory necrosis [37, 39]. Therefore, the involvement of pyroptosis in the pathogenesis of schizophrenia could not be ruled out. Surprisingly, we did not find changes in the expression of miR-3653-3p, NLRP3, Caspase 1, and IL-1β in 8 patients with schizophrenia after 12 weeks of treatment. It may be a bias caused by the small number of completed visits, and our subjects had high heterogeneity in drug use and overall disease course. This study was only verified in clinical samples. Follow-up could be continued in the future, or a comparative study could be conducted between the untreated schizophrenia and chronic schizophrenia patients to detect the expression levels of miR-3653-3p, NLRP3, caspase 1, and IL-1β. Furthermore, the relationship between miR-3653-3p/NLRP3/caspase 1/IL-1β and the pathogenesis of schizophrenia was further verified by in vitro and in vivo experiments.

Severe psychotic symptoms and impaired social functioning are the main characteristics of acute schizophrenia [1], but so far, no stable biomarkers in peripheral blood have been identified. We are curious about this and the starting point for us to explore key small molecular biomarkers in peripheral blood. IL-1β may be a marker of the chronic course of schizophrenia or a target for clinical intervention. Due to their good stability and signal communication characteristics, miRNAs are still expected as biomarkers for neuropsychiatric disorders. However, due to poor data normalization, the interpretation of miRNA function and its exploration as biomarkers are still in the early stage [19].

## Conclusion

We focus on patients with schizophrenia in emergency hospitalization, which contains almost all the patients with schizophrenia needed for treatment. The expression of miR-3653-3p, NLRP3, caspase 1, and IL-1β in schizophrenia does show interconnection. Maybe they are related to the chronic course of schizophrenia or the negative symptoms of schizophrenia. In the future, the relationship between miR-3653-3p and NLRP3, caspase 1, and IL-1β in patients with schizophrenia is worthy of in-depth research and further validating the potential of miR-3653-3p, NLRP3, caspase 1, and IL-1β as a stable biomarker for schizophrenia in diagnostic experiments. Also, we need to verify the role of pyroptosis in the pathological mechanisms of schizophrenia.

### Supplementary Information

**Additional file 1.** Primer sequence.

## Data Availability

The data used and analyzed during the current article are available. Please visit the links below: https://data.mendeley.com/datasets/md6g8f7xgj/1.
