# Supplementary material for: Abnormal expression of miR-3653-3p, caspase 1, IL-1β in peripheral blood of schizophrenia
Source: BMC Psychiatry. 2023 Nov 9;23:822. doi: 10.1186/s12888-023-05182-0 (PMC10633926; doi:10.1186/s12888-023-05182-0)
Supplement: Supplementary file 1 — Additional file 1. Primer sequence. [file 12888_2023_5182_MOESM1_ESM.docx]

**Primer sequence**

| **ACTIN** | F：GGCCAACCGCGAGAAGATGAC R：GGATAGCACAGCCTGGATAGCAAC |
| --- | --- |
| **IL-1β mRNA** | F：GGACAGGATATGGAGCAACAAGTGG R：TCATCTTTCAACACGCAGGACAGG |
| **Gas-D mRNA** | F：ACAGCTCCAGCACCTCAATGAATG R：GCACCTCAGTCACCACGTACAC |
| **Caspase1 mRNA** | F：GCAGATGCCCACCACTGAAAGAG R：TCCCACACTCCCGACCATACAC |
| **hsa-miR-3653-3p** | F：5‘--GTCGTATCCAGTGCGTGTCGTGGAGTCGGCAATTGCACTGGATACGACCTTCAGT--3’  f：GGGGCTAAGAAGTTGAC  r：CAGTGCGTGTCGTGGAGT" |
| **U6** | F：CTCGCTTCGGCAGCACA R：AACGCTTCACGAATTTGCGT |
| **NLRP3 mRNA** | F：AGGGATGAGAGTGTTGTGTGAAACG R：GCTTCTGGTTGCTGCTGAGGAC |
